# A dehydrin gene isolated from feral olive enhances drought tolerance in *Arabidopsis* transgenic plants

**DOI:** 10.3389/fpls.2015.00392

**Published:** 2015-06-30

**Authors:** Adriana Chiappetta, Antonella Muto, Leonardo Bruno, Magdalena Woloszynska, Mieke Van Lijsebettens, Maria B. Bitonti

**Affiliations:** ^1^Laboratory of Plant Biology, Department of Biology, Ecology and Earth Science, University of CalabriaCosenza, Italy; ^2^Department of Plant Systems Biology, VIB, Ghent UniversityGhent, Belgium; ^3^Department of Plant Biotechnology and Bioinformatics, Ghent UniversityGhent, Belgium

**Keywords:** dehydrins, drought tolerance, gene expression, *oleaster*, green fluorescent protein

## Abstract

Dehydrins belong to a protein family whose expression may be induced or enhanced by developmental process and environmental stresses that lead to cell dehydration. A dehydrin gene named *OesDHN* was isolated and characterized from *oleaster* (*Olea europaea* L. subsp. *europaea*, var. *sylvestris*), the wild form of olive. To elucidate the contribution of *OesDHN* in the development of drought tolerance, its expression levels were investigated in *oleaster* plants during development and under drought stress condition. The involvement of *OesDHN* in plant stress response was also evaluated in *Arabidopsis* transgenic lines, engineered to overexpress this gene, and exposed to a controlled mild osmotic stress. *OesDHN* expression was found to be modulated during development and induced under mild drought stress in *oleaster* plants. In addition, the *Arabidopsis* transgenic plants showed a better tolerance to osmotic stress than wild-type plants. The results demonstrated that *Oes*DHN expression is induced by drought stress and is able to confer osmotic stress tolerance. We suggest a role for *OesDHN*, as a putative functional marker of plant stress tolerance.

## Introduction

Due to their sessile organization, plants are exposed to wide diurnal, seasonal, and stochastic fluctuations. Consequently, plants developed a large spectrum of molecular programs to sense environmental changes rapidly and adapt accordingly (Sakuma et al., 2006; Meehl et al., 2007; Ahuja et al., 2010). However, despite this adaptive plasticity, in the last century the continuous anthropogenic impact and the ongoing climate changes negatively affected plant development and productivity (Zohary and Spiegel-Roy, 1975; Ceccarelli et al., 2007). Therefore, there is an increasing requirement to develop varieties with enhanced tolerance to multiple stresses, because in nature plants are often simultaneously exposed to several perturbations factors.

Understanding the reprogramming events that plants put in place in response to environment changes is of longstanding interest for plant biologist. Among defense mechanisms, a number of regulatory and/or protective proteins involved in plant tolerance to different stresses, have been identified (Choi et al., 1999; Skinner et al., 2005; Svensson et al., 2006; Yamaguchi-Shinozaki and Shinozaki, 2006). Dehydrin proteins (DHNs) belong to these common protectants and their presence has been observed in several independent studies on drought, and salinity stresses as well as on cold acclimation and after abscisic acid (ABA) treatment (Close et al., 1989; Porat et al., 2004; Rorat et al., 2006; Tripepi et al., 2011). Therefore, they are referred also as RAB (responsive to ABA) and COR cold-regulated proteins (Close, 1996; Nylander et al., 2001).

DHNs are the group II, D 11 family of highly hydrophilic proteins, known as LEA (Late Embryogenesis Abundant), that accumulate in the late stages of embryogenesis, when water content in seeds declines, or in response to various stressors (Dure, 1993; Close, 1996, 1997; Battaglia et al., 2008). DHNs are intrinsically unstructured proteins and their molecular mass ranges from 9 to 70 kilo dalton (kD) (Receveur-Bréchot et al., 2005; Graether and Boddington, 2014). Commonly, DHNs contain several conserved/identifiable motifs: the Y-segment, characterized by the consensus motif (T/V)D(E/Q)YGNP that is located near the N-terminus, the S-segment consisting of 5–7 Ser residues and the Lys-rich K-segment, usually located near the C-terminus, with an EKKGIMDIKEKLPG and (Q/E)K(P/A)G(M/L)LDKIK(A/Q)(K/M)(I/L)PG motif in angiosperms and gymnosperms, respectively (Close et al., 1995; Allagulova et al., 2003; Receveur-Bréchot et al., 2005; Graether and Boddington, 2014). Recently, three dehydrin genes with the absence of a tipical K-segment were identified and characterized in *Pinus pinaster* (Perdiguero et al., 2014). Several others motifs have been suggested for DHNs, but their conservation and ubiquity have not yet been defined (Perdiguero et al., 2012; Graether and Boddington, 2014). On the basis of the number of Y-, S-, and K-segments that they individually contain, DHNs are classified into five subclasses: YnSK2, Kn, SKn, Y2Kn, and KnS (Mundy and Chua, 1988).

In plants DHNs are expressed in different tissues and organs (Rorat et al., 2004). In some cases DHNs activity is restricted to specific domains or cells within the organs, as in guard cells, meristematic cells or in pollen sacs (Nylander et al., 2001; Karlson et al., 2003; Rorat et al., 2004; Layton et al., 2010). At cellular level DHNs can be found commonly in the cytoplasm and the nucleus, but also at the level of plasmodesmata, mitochondria, chloroplast and near the plasma membrane (Danyluk et al., 1998; Hara et al., 2003; Graether and Boddington, 2014; Xu et al., 2014).

Phylogenetically widespread, *DHNs* genes have been identified in cyanobacteria, algae, mosses, liverworts, lycopods and are ubiquitous in both angiosperms and gymnosperms species (Close, 1996, 1997; Campbell and Close, 1997; Allagulova et al., 2003; Layton et al., 2010). In the *Arabidopsis* proteome database 10 proteins bearing the typical DHN structure and 6 proteins with a K-segment-like sequence have been identified (Close, 1996; Bies-Etheve et al., 2008; Hundertmark and Hincha, 2008). Multiple DHN members have been also identified in different unrelated species and recently, 54 dehydrin unigenes have been identified in the *Triticum* genome (Velasco et al., 2007; Wang et al., 2007, 2014; Tommasini et al., 2008; Liu et al., 2012; Perdiguero et al., 2014).

According to the suggested involvement of DHNs in abiotic stress response, in several plants *DHNs* genes are induced under drought, cold and salinity (Porat et al., 2004; Lee et al., 2005). In addition, heterologous expression of *DHNs* genes, in different species, including relevant agronomic one such as rice, is associated with drought, cold and salinity tolerance (Hara et al., 2003; Rorat et al., 2006; Choudhury et al., 2007; Brini et al., 2010). Therefore, the identification of new *DHNs* associated with drought tolerance may provide useful markers for the selection of drought-tolerant genotypes by either breeding or transformation technologies.

To meet such requirement, in the present work we isolated and characterized a *DHN* gene from the wild form of olive, commonly named *oleaster* (*Olea europaea* L. subsp. *europaea*, var. *sylvestris* (Hoffm et Link). *Oleaster* is a typical and important shrub of vegetation present in the Mediterranean area, a climate region characterized by rainy winters and dry summers, with low air humidity, high solar radiation and high rates of evapotranspiration. *Oleaster* is largely prevalent in this habitat and displays a high photosynthetic efficiency and drought tolerance, related to its tap-root system that allows a deep exploration of the soil and a maximization of water uptake (Bacchetta et al., 2003; Mulas et al., 2011).

The *oleaster* identified gene, named *OesDHN* (*Olea europaea* subsp. *europaea* var. *sylvestris DEHYDRIN*), encodes a SK_2_-typeDHN and its expression has been found to be up-regulated in *oleaster* plants exposed to drought conditions. To elucidate the contribution of *OesDHN* in the development of drought tolerance, stable transgenic lines of *Arabidopsis thaliana* overexpressing this gene have been also developed. We show that under a mild osmotic stress, these *Arabidopsis* transgenic plants exhibit increased drought tolerance. The role of *OesDHN*, as a putative functional marker of plant stress tolerance is discussed.

## Materials and methods

### Isolation and sequence analyses of *OesDHN*

The gene was identified in a cDNA library generated from leaves of *oleaster* plants, growing in open field in the Mediterranean area in South Italy (117 meter above sea level, 39° 30′ 52. 77″N; 15° 56′ 28. 83′E). Leaves were collected in the summer, after a prolonged (2 weeks) period of high temperature (35 ± 2°C). The cDNA library was generated using the SMART system and cloning the sequence (around 1.2 kb) in the pSPORT1 vector. The sequencing analysis was performed from 5′end. Generation and sequencing of the library was performed by Eurofins MWG GmbH cDNA Laboratory Fraunhoferstr (De) service.

### Genomic organization of *OesDHN*

Total genomic DNA (gDNA) was isolated from leaves of *oleaster* plants growing in open field. Samples were frozen in liquid nitrogen and processed according to CTAB extraction method (ctyltrimethylammonium bromide) (Murray and Thompson, 1980). Southern blots and hybridizations analysis were performed as described in Bruno et al. (2009).

Briefly, after extraction and purification, gDNA (10 μg) was digested overnight at 37°C with *EcoRV* and *XbaI* endonucleases (Promega, Italy), which do not cut in the probe. The digested DNA fragments were separated on agarose gel 0.8% (w/v) and blotted onto a nylon membrane Hybond-N+ (Amersham Pharmacia Biotech, Milan, Italy), using the Vacuum Blotting System (BioRad, Milan, Italy). Hybridization was performed with a specific probe, corresponding to the *OesDHN* ORF (Open Reading Frame), labeled with DIG-dNTPs (PCR Dig Probe Synthesis Kit; Roche Diagnostics, Monza, Italy). The detection was performed with nitro blue tetrazolium chloride (NBT) and 5-bromo-4-chloro-3-indolyl phosphate (BCIP) mixture, following the manufacturer's instructions (Roche, Monza, Italy) and hybridization signals were scanned with a computer (Umax Speed II).

The gDNA was also used in order to evaluate the putative presence of introns, using the following primers: FwORF*OesDHN5′-ATGGCGGAGGAGGGACCCGTC-3′;* BwORF*OesDHN: 5′-TTAGTGGCATGCCCCCTCCTT-3′.*

### Isolation of the 5′-flanking region of *OesDHN*

The *OesDHN* promoter region was obtained by using the “Universal GenomeWalker™ kit” (Clontech, Diatech lab line, Milan, Italy) with some modifications. The purified gDNA was digested with four different blunt-ended restriction enzymes (*DraI*, *EcoRV*, *Pvu II*, *Stu I*) and the products were purified and ligated, separately, to “Genome Walker adaptors,” in order to produce four libraries. Two gene specific primers were used for the amplification of the promoter region. For the primary PCR (PCRI) were used: an AP1 primer (outer Adapter Primer), provided by the kit, a GSP1 primer (Gene Specific Primer 1; *5′-TTTCTTCTTCCTTGTGCTCCGGTTCACA-3′*) and the ligated products as template. The thermal cycling program was 94°C for 25 s and 72°C for 3 min; 7 cycles were used followed by 32 cycles of 94°C for 25 s and 67°C for 3 min, with a final extension at 67°C for 3 min. The products of the PCRI were then diluted and used as template for the secondary PCR (PCRII), using an AP2 primer (nested Adapter Primer 2) provided by the kit, and a GSP2 primer (Gene Specific Primer 2; *5′-CCC TGAAATCAAACAACCCACGGTCCT-3′*). For the PCRII the used parameters were 5 cycles of 94°C for 25s and 72°C for 3 min, followed by 20 cycles of 94°C for 25s and 67°C for 3 min, with a final extension at 67°C for 7 min. PCR amplifications were performed on a Step One machine (Applied Biosystems, Monza, Italy) and products were sequenced by GENELab laboratory (ENEA, Rome).

### Alignments and phylogenetics analysis

*Oes*DHN amino acid sequence was obtained using the ExPASy Proteomic Tools (http://www.expasy.org/tools/dna.html) program. The analysis and the comparison of *Oes*DHN deduced amino acid sequence with other dehydrin proteins were performed with blastp (Standard Protein-Protein BLAST) (http://blast.ncbi.nlm.nih.gov/Blast.cgi?PROGRAM=blastp&PAGE_TYPE=BlastSearch&LINK_LOC=blasthome) program. *Oes*DHN sequence was aligned with other plant dehydrins using the ClustalW2 program (http://www.ebi.ac.uk/Tools/msa/clustalw2/). A phylogenetic tree was generated by the MEGA4 software (Molecular Evolutionary Genetics Analysis) (Tamura et al., 2007) with bootstrap values obtained from 1000 replications and using the listed protein sequence: *Arabidopsis thaliana* COR47 (P31168), *Arabidopsis thaliana* ERD10 (P42759), *Arabidopsis thaliana* ERD14 (P42763), *Arabidopsis thaliana* DHLEA or PAP310, (Q96261), *Arabidopsis* thaliana Q9SLJ2 (At1g54410), *Arabidopsis thaliana* Q9SVE4 (75313932), *Arabidopsis thaliana* Q9T022, *Arabidopsis thaliana* RAB18 (P30185), *Arabidopsis thaliana* XERO1 (P42758), *Arabidopsis thaliana* XERO2 (P42758), *Avicennia marina* AmDHN1 (A8CVF3), *Brassica juncea* DHN2 (ABD95986), *Brassica oleracea* BOPC34 (CAA64428), *Citrus trifoliata* COR11 (AAA99963.1), *Craterostigma* plantagineum (P22238), *Glycine max* MAT9 (AAA33992), *Hordeum vulgare* subsp. *vulgare* DHN1(P12951), *Hordeum vulgare* subsp. *vulgare* DHN5 (AAF01693), *Hordeum vulgare* subsp. *vulgare* PAF93 (CAA58875), *Jatropha curcas Jc*DHN-1 (ADT65200), *Medicago sativa* G2 (AEV52291), *Medicago sativa* subsp. *falcata* CAS18 (AAA21185), *Opuntia streptacantha* DHN1 (AEI52546), *Oryza sativa Japonica* WSI724 (BAA05539), *Oryza sativa Japonica* DH16D or RAB16D (P22913), *Pisum sativum* DHN2 (CAA44788), *Pisum sativum* dhn-cog (CAA78515), *Populus alba x Populus glandulosa Po*DHN (ABH11546), *Prunus persica* DHN1 (P28639), *Prunus persica* PCA60 (AAC49657), *Rhododendron catawbiense* DHN-5 (ACB41781), *Solanum sogarandinum* DHN24 (AAP44575), *Triticum aestivum* COR410 (P46524), *Triticum aestivum* CS120(P46525), *Triticum aestivum* CS66 (P46526), *Triticum aestivum* RAB15 or DHR15 (Q00742), *Vitis vinifera* DHN1b (526118232), *Zea mays* COR410 *Zea mays* (226532837), *Zea mays* DEHYDRIN 13 (EU962627.1), *Zea mays* DHN1 (18963), *Zea mays* lipase DHN2 (L35913.1), *Zea mays* Put. unch. protein (219363418), *Zea mays* RAB17 (18963). Analysis of targeting sequences was performed using PSORT (http://psort.nibb.ac.jp/) and Wolf PSORT (http://wolfpsort.seq.cbrc.jp/) programs.

### Drought assay in OLEASTER plants

Drought stress was performed on 2 years old *oleaster* plants (*n* = 10) obtained through micropropagation and grown in pots on standard sandy loam soil and daily irrigation. Drought condition was applied by depriving progressively the plants of water content as described by Tommasini et al. (2008). The soil water content (SWC) was calculated relative to 100% water capacity of soil by weighing plant at different times. We considered the 100% SWC the weight of plants wetted until saturation, covered with parafilm at the soil surface and drained for 1 h. These samples were used as control.

For drought stress samples were collected when SWC values were approximatively the 45%, the mild drought stress, and 37%, the high drought stress, reached after 14 and 35 days, respectively.

### qRT-PCR in OLEASTER plants

qRT-PCR analysis was performed: (i) on *oleaster* plants (*n* = 5) growing in open field in the Mediterranean area, in South of Italy (117 meter above sea level, 39° 30′ 52. 77″N; 15° 56′ 28. 83′E). In this case different organs were analyzed: apical vegetative shoot, young (1–2 cm) and adult (6–8 cm) leaves, stem (harvested at the resumption of the vegetative growth) and green drupes; (ii) on mature leaves (2.5 cm) and stem of 2 years old *oleaster* plants, (*n* = 10), exposed to drought assay.

For both groups, excised organs were immediately frozen. Total RNA was isolated from 100 mg of *oleaster* samples, using the RNeasy Plant Mini kit (Qiagen, Hilden, Germany), as previously described by Bruno et al. (2009). One microgram of total RNA was used to generate the single-strand cDNA by the SuperScript III Reverse Transcriptase and the oligodT(20), following manufacturer's instructions (Invitrogen, Milan, Italy). The cDNA concentration was determined by the NanoDrop Spectrophotometer ND-1000.

A Step One single color thermocycler (Applied Biosystems, Monza, Italy) with Power SYBR Green PCRMaster Mix 2X (Applied Biosystem, Monza, Italy) (Cat. no. 4368702) were utilized. The primer sets used, the *OesDHN* FW 5′-AAG GAG AAG CTC CCT GGG TA -3′ and *OesDHN* BW 5′-AAA CCA CCA AAG AAG AAA TCA AA -3′ were designed using the Primer3 (http://primer3.ut.ee/) program, according to Yokoyama and Nishitani (2001). The olive 18S histone was used as a normalization control. The *Oes18S* primer sequences were *Oes18S* FW 5′-CAG CCT TCA ATG ATC GGA AT-3′ and *Oes18S* BW 5′GCG CTG TAA TTT CCT TGC TC. Amplification reactions were prepared in a final volume of 25 ml by adding 12.5 ml of the iTaq SYBR-Green Super Mix with ROX (Bio-Rad, Milan, Italy), 1 ml (0.4 mM) of primers, and 2 ml (25 ng) of cDNA. All reactions were run in triplicate, in 48-well reaction plates, and negative controls were set. The cycling parameters were performed as described in Bruno et al. (2014). The quantitative qRT-PCR data were analyzed using the Step One Software 2.0 (Applied Biosystems, Monza, Italy) with the 2^−ΔΔCT^ method (Livak and Schmittgen, 2001). The means of *OesDHN* expression levels were calculated from three biological repeats, obtained from three independent experiments.

### Histological analysis

Mature leaves (*n* = 15) collected from stressed and unstressed plants were fixed in 3% (w/v) paraformaldehyde and 0.5% (v/v) glutaraldehyde in PBS buffer (135 mM NaCl, 2.7 mM KCl, 1.5 mM KH2PO4, 8 mM K2HPO4 pH 7.3) for 3 h at 4°C. After washing in the same buffer, samples were dehydrated and embedded in Tecknovitt 8100 resin Heraeus Kulzer (Germany). Semi-thin sections (4 μm) were obtained using an ultracut microtome (Leica RM 2155, Milan, Italy) and stained with toluidine blue O 0.05% in distilled water.

The histological samples were analyzed by bright-field illumination (Leica DRMB, Milan, Italy) and images were taken with the digital camera Leica DFC 320 (Leica, Milan, Italy).

### Dehydrins immunolocalization

Dehydrins immunolocalization was performed as described in Carjuzaa et al. (2008). Mature leaves (*n* = 15) were collected from stressed and unstressed plants and fixed in 2% (w/v) paraformaldehyde and 1% (v/v) glutaraldehyde in 0.1 M phosphate buffer, ph7.2 for 2 h at 4°C. Samples were washed with deionized water for 30 min, dehydrated and embedded in paraplast (Sigma-Aldrich, Milan, Italy). Afterwards, they were cut with an RM 2125 RT microtome (Leica, Milan, Italy) into 8 μm sections that were transferred to charged slides and incubated with anti-dehydrin primary antibody (LiStarFish, Milan, Italy) diluited 1:200 at 4°C overnight. Subsequently, sections were incubated with goat anti-rabbit IgG alkaline phosphate conjugate, (Calbiochem, Milan, Italy) diluted 1:100 for 4 h at 4°C. After washing, the sections were developed with a NBT (nitro blue tetrazolium) and BCIP (5-bromo-4-chloro-3-indolylphosphate) mixture, for 5 min, rinsed and immediately observed with a Leica DRMB microscope (Leica, Milan, Italy) equipped with a Leica DFC 320 camera. To verify the effectiveness of the immunolocalization technique, sections were processed with the omission of the primary anti-dehydrin antibody.

### Recombinant constructs using the gateway technology

*OesDHN* cDNA, obtained as previously described, was cloned into the pDONOR221(Invitrogen) plasmid using the Gateway method. The sequence captured as entry clone was recombined in both pK7WG2 and pKF7WG2/pK7WGF2 vectors (Karimi et al., 2002) for *OesDHN* overexpression analysis and for its localization in N- and C- terminal with the green fluorescent protein (GFP) fusion, respectively, under the control of the cauliflower mosaic virus 35Spromoter.

For Gateway the following primers were used: FWattB1ORFnoSTOP:5′GGGGACAAGTTTGTACAAAAAAGCAGGCTTCATGGCGGAGGAGGGACCCGTC-3′; BWattB2plusSTOP:5′-GGGGACCACTTTGTACAAGAAAGCTGGGTCTTAGTGGCATGCCCCCTCCTT-3′; BWattB2noSTOP:5′-GGGGACCACTTTGTACAAGAAAGCTGGGTCGTGGCATGCCCCCTCCTT-3′; FWattB1 5′-GGGGACAAGTTTGTACAAAAAAGCAGGCT-3; BWattB25′-GGGGACCACTTTGTACAAGAAAGCTGGGT-3′.

**Figure 1:**
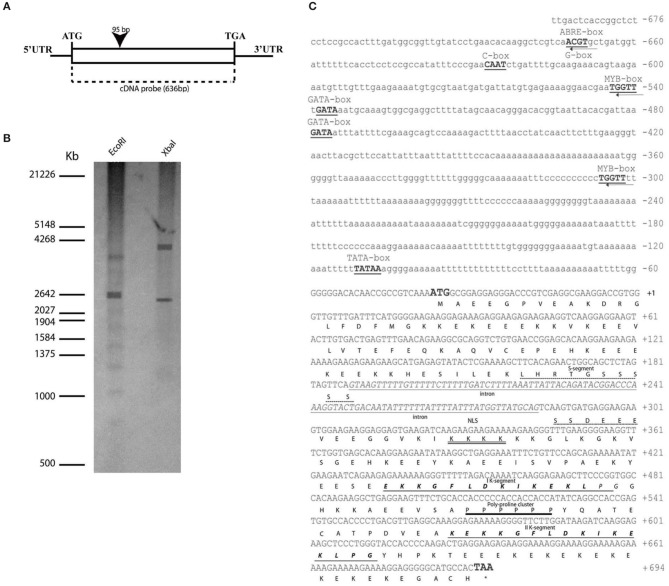
***OesDHN*** **genomic organization. (A)** Scheme of *OesDHN* gene. The black rectangle indicates intron position with respect to the cDNA. Start and stop codons are typed. **(C)** Deduced nucleotides of *OesDHN* cDNA and its 5′ upstream region and features of *Oes*DHN protein. The ATG start and TAA stop codons are indicated in bold; the stop codon position is indicated by the asterisk. The intron sequence, including the putative TATA-box (bold) is highlighted. The putative ABRE, G-box, MYB, GATA, CAAT-box elements are also indicated. Predicted amino acids are shown in one letter code. The S-segment is shown with an interrupted line, and the two K-segments are shown in bold italics and underlined with a single line. The putative NLS (nuclear localization signal) is double underlined and the poly-proline cluster is shown with one single line. The intronic region is represented with italic lowercases. **(B)** Southern blot analysis. The *oleaster* gDNA was digested with *EcoRI* and *XbaI* and hybridized with the cDNA probe, indicated in **(A)**. The molecular weights of a co-migrating DNA marker are expressed in kilo base pairs (Kb). PR, promoter region; UTR, Untranslated region. Bar 200 bp **(A)**.

The constructs are schematized in Figure 1S. They were sequenced by GENELab (ENEA, Rome), transferred into *Agrobacterium tumefaciens* (GV3101) by heat shock and used to transform *Arabidopsis thaliana* accession *Col-*0 plants by floral dipping (Clough and Bent, 1998; Davis et al., 2009).

### Selection and qRT-PCR of arabidopsis transgenic lines

*Arabidopsis Col-*0 seedlings were grown *in vitro* on Murashige and Skoog (MS) medium (Murashige and Skoog, 1962) plus vitamins (Sigma, Milan, Italy), supplemented with 0.1 g/l of myo-inositol, 0.5 g/l of 2-N-morpholine ethane sulphonic acid (MES), 10 g/l sucrose and 8 g/l agar, pH of 5.7. Plants were grown under 16 h day at 22°C and 8 h night at 18°C regime, light intensity was 100 μmol m^−2^ s^−1^ and the relative humidity (RH) was 65–70%.

*Arabidopsis* transgenic lines were selected on a MS medium plus vitamins, supplemented with 50 μg/ml Kanamycin (Km), 250 μg/ml di Carbenocillin (Cb) and 50 μg/ml Nystatin (Ns) or with only 50 μg/ml Km.

The number of T-DNA loci for each line was determined by chi-square statistical method. The presence of the transgene, in T3 homozygous lines with 1 T-DNA locus, was confirmed through qRT-PCR, using at least three pairs of gene specific primers (*FW1OesDHNRT:5′-CTGGTGAGCACAAGGAAGAA-3; BW1OesDHNRT: 5′TGGTGCAGAAACTTCCTCAG-3′; FW2OesDHNRT:5′-CTGAGGAAGTTTCTGCACCA-3′; BW2OesDHNRT: 5′-TCTCCTTTGCCTCAACGTC-3′; FW3OesDHNRT:5′-TGGGTTGTTTGATTTCATGG-3; BW3OesDHNRT: 5′-CTTCCTTGTGCTCCGGTT-3′*, located at different points of the ORF and designed by GenScript Real-time Primer Design program (https://www.genscript.com/ssl-bin/app/primer).

The qRT-PCR was performed by the LightCycler 480 machine (Roche Diagnostics) according to the manual and the parameters of annealing and extension were: 45 cycles of 95°C for 10 min, 60°C for 50 s, 72°C for 50 s. The reaction mix was prepared by using a robotic platform (JANUS, Perkin Elmer, Massachusetts, USA). The melting curves were performed to analyze the specificity of primers. Normalization was performed using the followed housekeeping genes: SAND family (AT2G28390), TIP41-like (AT4G34270), UBC (AT5G25760) and PP2A subunits (AT1G13320) and primers (*FWSAND family: 5′-AACTCTATGCAGCATTTGATCCACT-3′; BWSAND family: 5′-TGATTGCATATCTTTATCGCCATC-3′; FWTIP41-like:5′ GTGAAAACTGTTGGAGAGAAGCAA-3′; BWTIP41-like: 5′-TCAACTGGATACCCTTTCGCA-3′*; FWPP2A subunit: *5′-AACTCTATGCAGCATTTGATCCACT-3′; BWPP2A subunit: 5′-TGATTGCATATCTTTATCGCCATC-3′; FWUBC: 5′-CTGCGACTCAG^GGAATCTTCTAA-3′; BWUBC: 5′-TTGTGCCATTGAATTGAACCC-3′;* (Czechowski et al., 2005). The results were analyzed using the LightCycler software with the 2^−ΔΔCT^ method (Livak and Schmittgen, 2001). The Ct-values were calculated from three biological replicates, obtained from three independent experiments.

### Osmotic assay in arabidopsis 35S::*OesDHN* transgenic plants

Sixteen seedlings of transgenic line, 35S::*OesDHN* B1-2, with high transgene expression were germinated on MS medium containing mannitol 25 mM (Sigma, Milan, Italy) and leaves were harvested after 22 days. Leaves were dissected under a stereomicroscope, placed in square plates on 1% (w/v) plant tissue agar, and then photographed. Length and leaf area were measured by ImageJ program (rsbweb.nih.gov/ /ijdownload.html) and mean values of three independent experiments were subjected to statistical analysis.

### Confocal microscopy

Confocal analysis were performed on 6 old days *Arabidopsis* seedlings of T3 35S::*OesDHN*:GFP line A 4-1, 35S::GFP:*OesDHN* line C11-5 and *Col*-0 plants grown *in vitro*, in vertical position and under continuous light regime. Samples were mounted in water and observed under a confocal microscope 100M Zeiss (Zeiss, Germany), equipped with the software package LSM 510 version 3.2. Excitation and emission wavelenghts used were 488-nm and 500–550-nm band-pass filter. Chlorophyll autofluorescence was imaged using 488 nm for excitation and 633 nm for emission. The green and red autofluorescence were collected in separate channels.

### Sequences deposition

The sequence obtained in this study was submitted to the GenBank with the following accession number: KR349290.

## Results

### Genomic organization of OesDHN and analysis of its 5′flanking region

*OesDHN* was identified from a cDNA full length library, generated from total RNA of *oleaster* leaves harvested in summer, after a prolonged periods (2 weeks) of high temperatures (*T*_Max_ = 35 ± 2°C; *T*_Min_ = 26.5 ± 2°C). The generated 1200 unigenes were functionally annotated based to the best BLASTX hit, against the NCBI database with a cut-off of *E* < 10–15. A large number of genes (40%), corresponding to stress-responsive gene were identified, and among these we selected a member of *DHN* gene family.

The full-length cDNA of *OesDHN* was 1207 bp long and showed an ORF of 636 and a 31 bp 5′-UTR and a 540 bp 3′-UTR regions. Comparison of genomic and cDNA sequence and T BLASTX analysis showed that the *OesDHN* gene contained a single intron of 95 bp (Figure 1A).

To determine the copy number of *OesDHN* gene in the *oleaster* genome we performed a southern blot analysis using the 636 bp cDNA fragment as probe. After gDNA digestion by *EcoRI* and *XbaI* the hybridization pattern showed two cross-hybridizing bands, suggesting that at least two copies are present in the *oleaster* genome (Figure 1B).

We also isolated a 676 bp *OesDHN* promoter region. Analysis of its sequence, using the “PLACE” and “PlantCARE” databases (Lescot et al., 2002), revealed the presence of a TATA-box sequence, located 69 bp upstream of the translational start site (TSS) (Figure 1C). As reported for many dehydratation-responsive genes (Zhu, 2002), the 5′ region contained putative *cis*-regulatory sequences, such as an ABA-responsive element (ACGT sequences) also named ABRE and a G-box (CACGTT) element identified at the -631 bp position, and two MYB-binding consensus sequences (WAACCA) located at -502 and -264 bp upstream the TSS, respectively Furthermore, two GATA boxes regulatory elements, with homology to those identified in light-responsive genes, were detected at the position -497 and -438 bp. Finally, a CAAT-box (C-box), a common *cis*-enhancer promoter region, was identified at the position -586 (Figure 1C).

### Characterization and phylogenetic analysis of deduced *OesDHN*

*OesDHN* encodes a putative protein of 211 amino acids, with a predicted molecular weight of 241 kDa and an isoelectric point of 53 (Geneious 7.1.5). The multiple amino acid sequence alignment revealed that *Oes*DHN shares all common features of plant DHNs family (Figure 1C). Namely, a conserved region with serine residues, the S-segment, was located between the residues 66 and 80 and two lysine-rich consensus motifs, the K-segments, were present between the residues 126–140 and 171–185, respectively, closed to the C-terminal region. As showed for other plant DHNs, a putative nuclear localization signal peptide (NLS), identified by the KKKK sequence, was located between the 90–93 residues downstream the S segment (Figure 1C). Finally, a poly-proline cluster, an important component of intrinsically unstructured proteins (Rath et al., 2005; Harauz and Libich, 2009), was located at residues 151–156 between the K segments (Figure 1C).

**Figure 2:**
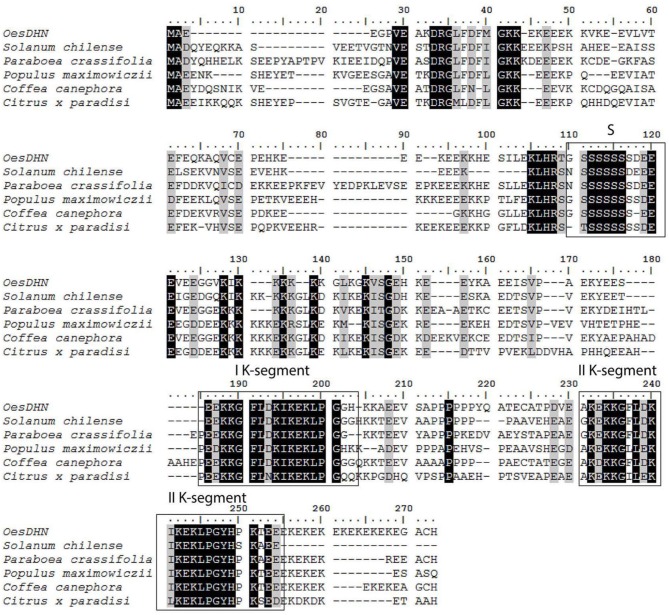
**Sequence alignment of** ***Oes*****DHN protein with homologs from other plant species performed with the NCBI database**. Accession numbers are given in parentheses: *Solanum chilense* (ADQ73953.1), *Paraboea crassifolia* (AAF014652.2), *Populus maximowiczii* (ABS12346.1), *Coffea canephora* (ABC68275.1), and *Citrus x paradise* (AAN78125.1). The multiple amino acid sequence alignment reveals the common features of *Oes*DHN with plant dehydrin family, such as the S- and K- segment, that are boxed.

The *Oes*DHN deduced protein exhibited a significant degree of amino acid sequence identity with known homologs, i.e., 63% identity and 73% similarity with DHN of *Solanum chilense*, by 60% identity and 67% similarity with BDN1 of *Paraboea crassifolia*, 50% identity and 70% similarity with *Populus maximowiczii*, 56% identity and 68% similarity with *Coffea canephora*, and 52% identity and 66% similarity with DHN in *Citrus x paradise* (50%) (Figure 2).

**Figure 3:**
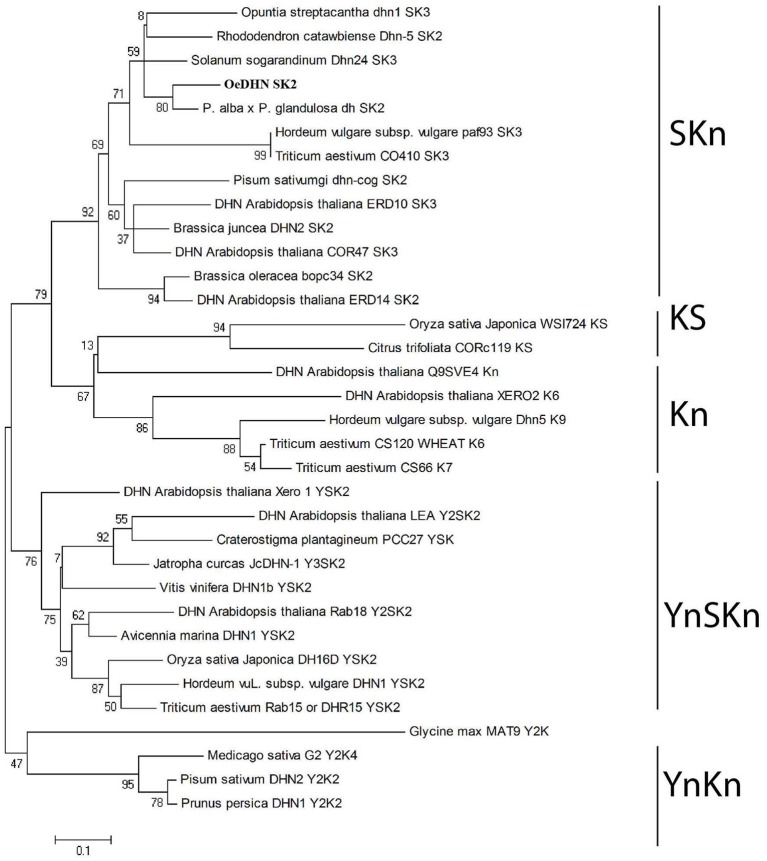
**Phylogenetic tree of DHN proteins**. The neighbor-joining tree was generated using the MEGA4 (www.megasoftware.net) version 5.0 software, with bootstrap value obtained from 1000 replications.

A phylogenetic tree, using YnSKn, SKn, Kn, YnKn, and KnS- type DHNs indicated that *Oes*DHN clustered together with SK_n_-type dehydrins (Figure 3). Thus, the results of both homology and phylogenetic analyses suggest that *Oes*DHN is an SK_2_-type DHN.

### In OLEASTER plants *OesDHN* is modulated during development and induced under drought stress condition

**Figure 4:**
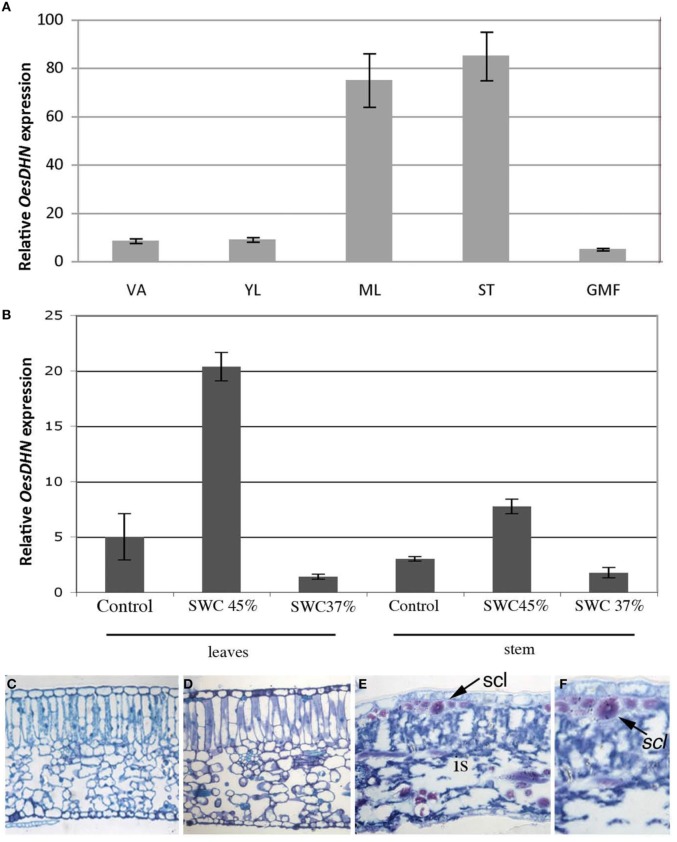
**(A)** Relative expression levels of *OesDHN*, estimated by qRT-PCR, in different organs of *Olea europaea* L. subsp. *europaea* var. *sylvestris* grown in open field and **(B)** grown in pots and exposed to a SWC of 45 and 37%. The levels of *OesDHN* were normalized to *Oes18S* histone. Data shown are averages of three biological replicates, with error bars representing SD. **(C–F)** Cross sections of mature leaves (length 2.5 ± 0.3 cm) of *oleaster* plants grown in pots under control condition **(C)** and exposed to a soil water content (SWC) of 45% **(D)** and 37% **(E,F)**. VA, Vegetative apex; YL, young leaves; ML, mature leaves; ST, Stem; GMF, green mature fruit; is, intercellular spaces; scl, sclerenchyma fibers. Arrow indicates sclerenchyma fibers. Bars 25 μm **(C–E)**; 43 μm **(F)**.

Tissue specific *OesDHN* expression was analyzed through qRT-PCR in *oleaster* plants grown in open field (Figure 4A). *OesDHN* expression was observed in all examined tissues, although at different level. Indeed, transcript accumulation *OesDHN* was higher in mature leaves and stem than in young leaves and shoot apex and was lowest in green mature fruits (Figure 4A).

To test whether *OesDHN* expression could be induced in response to stress 2 years old *oleaster* plants growing in pots, in greenhouse, were exposed to mild [soil water content (SWC) = 45%] and high (SWC = 37%,) drought stress (Figures 4B–F). Gene expression level was evaluated in the mature leaves and stem of stressed vs. unstressed plants. A significant increase in transcript level was detected both in leaves (four-fold increase) and stem (two-fold increase) under mild stress condition while a reduction was monitored in the organs of plants exposed to high drought stress compared to those of unstressed plants (Figure 4B).

Leaf anatomy was analyzed in stressed vs. unstressed plants as a parameter to monitor the effects of water deficit at the tissue organization level (Figures 4C–F). Typically, in control plant mature leaf (length 2.5 ± 0.3 cm) exhibited a dorsoventral anatomy with mesophyll formed by a two-layered palisade tissue and a well-developed spongious tissue (Figure 4C). No significant alterations in leaf structure were observed when a mild drought stress (45% SWC) was imposed (Figure 4D). In contrast, in the leaves of plants exposed to high drought stress (37% SWC) cells featured clearly plasmolyzed and mesophyll exhibited wide intercellular spaces although leaf collapse was prevented thanks to the presence of numerous sclerenchyma fibers (Figures 4E,F).

All together these results demonstrated that *OesDHN* expression is modulated during development and induced under mild drought stress.

Immuocytological assay allowed us to verify that DHNs accumulate under drought stress in *oleaster* leaves and exhibit both a cytosol and nuclear localization (Figure 2S).

### Overexpression of *OesDHN* improves osmotic stress tolerance in arabidopsis transgenic lines

**Figure 5:**
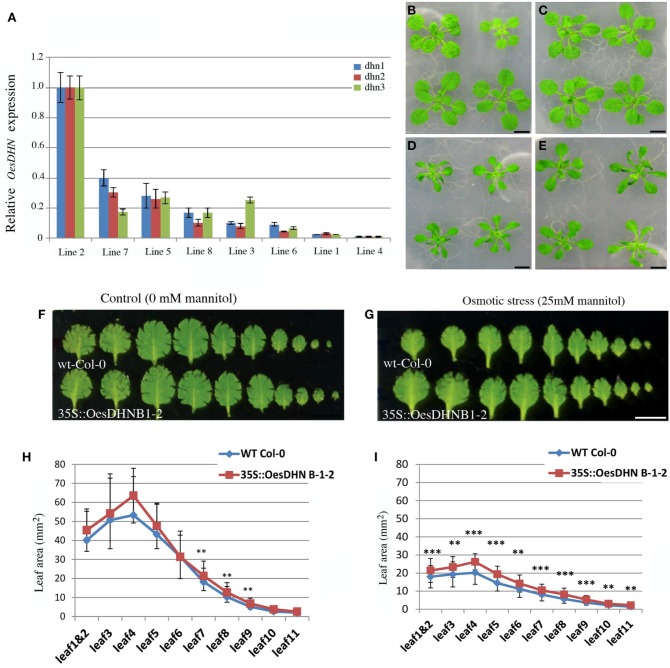
**(A)** Relative expression levels of *OesDHN*, estimated by qRT-PCR, in 35S::*OesDHN* lines. The levels of *OesDHN* were normalized to *TIP41* and *PP2* housekeeping genes. Data shown are averages of three biological replicates, with error bars representing SD. **(B–E)** Effects of mannitol concentration on the vegetative growth of *Col*-0 **(B,D)** and 35S::*OesDHN* B 1-2 line **(C,E)** plants grown under control conditions **(B,C)** and a controlled mild osmotic stress **(D,E)** for 22 days. Leaf series of *Col*-0 and 35S::*OesDHN* B 1-2 line plants grown under control conditions **(F)** and a controlled mild osmotic stress **(G)** for 22 days. Leaf area (mm^2^) of *Col*-0 and 35S::*OesDHN* B 1-2 line plants grown under control conditions **(H)** and a controlled mild osmotic stress **(I)** for 22 days. The measurements were carried out using the program Image J and “^*^” indicates the significance. (*t*-test ^*^p < 0.05 is significant; ^**^p < 0.01 is very significant; ^***^p < 0.001 is highly significant). dhn1, dhn2, dhn3 indicate three pairs of gene specific primers located in different points of the *OesDNH* ORF. Bars 1 cm **(B–G)**.

To confirm the involvement of *OesDHN* in plant stress response, *Arabidopsis* 35S::*OesDHN* lines were generated and the effects of a controlled mild osmotic stress (Skirycz et al., 2010) were analyzed using *Arabidopsis* 35S::*OesDHN* T3 B1-2 and 35S::*OesDHN* T3 B9-7 transgenic lines, with highest transgene expression (Figure 5A). We report the results obtained with the 35S::*OesDHN* T3 B1-2 (Figures 5B–E).

Leaf series were made of seedling rosettes, 22 days after germination and before bolting and leaf area was measured (Figures 5F–I). Under normal conditions, no difference in leaf area was observed between the transgenic line and wild type with the exception of leaf 7, 8, and 9 (Figures 5F,H). Under mannitol stress 25 mM leaf size was constantly and significantly higher in transgenic line compared to the control (Figures 5D,E,G,I). The strongest effects were observed on 4 and 5 leaves that in 35S::*OesDHN* B 1-2 line were respectively 15 and 10%, larger then control (Figures 5G,I).

These results suggest that the growth of *Arabidopsis* 35S::*OesDHN* plants, under a mild osmotic stress, is less inhibited by mannitol treatment as compared to that of the wild type.

No significant difference were observed on the primary root length and lateral roots formation in both genotypes grown in presence of mannitol (data not shown).

### *OesDHN* exhibits a nuclear localization

**Figure 6:**
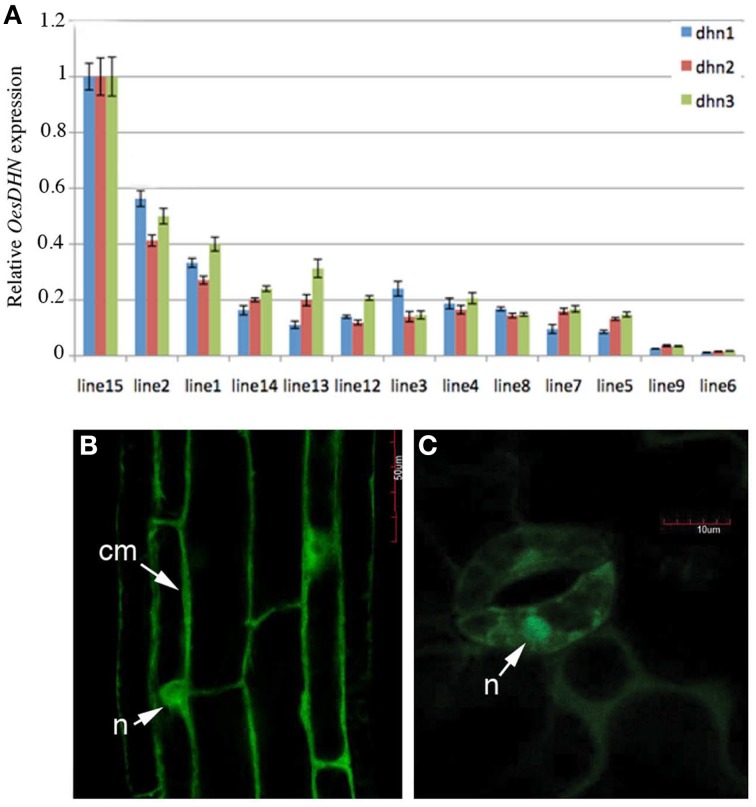
**(A)** Relative expression levels of *OesDHN*, estimated by qRT-PCR, in 35S::OesDHN:GFP lines. The levels of *OesDHN* were normalized to *TIP41 and PP2* housekeeping genes. Data shown are averages of three biological replicates, with error bars representing SD. **(B,C)** subcellular localization of the *Oes*DHN protein fused in N and C-terminal GFP under the control of the CaMV35S promoter in stable T2 35S::*OesDHN*:GFP line A 4-1, 35S::GFP:*OesDHN* line C11-5 lines of *Arabidopsis* with one T-DNA locus. **(B)** Root maturation zone; **(C)** Guard cells of stomata, located on the abaxial side of leaves. cm, cell membrane; n, nucleus. dhn1, dhn2, dhn3 indicate three pairs of gene specific primers located in different points of the *OesDNH* ORF.

To define the subcellular localization of *Oes*DHN, *Arabidopsis* 35S::GFP:*OesDHN* and 35S::*OesDHN:*GFP T3 lines were generated and *Oes*DHN localization was performed by confocal microscope on 8 old days seedlings of the 35S::*OesDHN*:GFP A 4-1, 35S::GFP:*OesDHN* C11-5 lines, exhibiting the highest levels of gene expression (Figure 6A).

*Oes*DHN presence was observed in the cytosol near the putative cell membrane and clearly in the nucleus in the epidermis of primary roots and guard cells of stomata (Figures 6B,C). This last result is consistent with *in silico* characterization, indicating the presence of a nuclear localization signal peptide.

## Discussion

Understanding the molecular mechanisms that allow plants to cope with abiotic stress can help to manipulate plant breeding with the aim to improve their tolerance and adaptation and preserve their productivity. Currently, drought is among major abiotic stresses impacting on plant development and productivity. Therefore, in the present study we focused our attention on DHNs, a protein family known to play a protective role during dehydration process induced by either developmental cues, such as embryo maturation, pollen development and bud dormancy, or different environmental factors including drought, salinity heat and cold (Gilmour et al., 1992; Nylander et al., 2001; Ali-Benali et al., 2005; Brini et al., 2007, 2010; Hanin et al., 2011; Tripepi et al., 2011). So far, the precise role of DHNs in plants has not yet been fully defined. At this respect, we may recall that due to their intrinsically disordered structure DHNs are prevented from denaturation and exhibit high flexibility, structural adaptability and extended conformations (Tompa, 2002; Bokor et al., 2005; Mouillon et al., 2008; Cuevas-Velazquez et al., 2014).

Consistently with these characteristics, DHNs have been found to bind to divalent cations and other macromolecules, such as proteins, nucleic acid and negatively charged lipids (Campbell and Close, 1997; Waterer et al., 2010). Based on all these features, numerous activities have been suggested for DHNs, including buffering water, sequestering ions, acting as space filler, stabilizing membranes and cellular structural components, preventing enzyme inactivation by protein-protein interaction or acting as chaperones and molecular shields in response to the different stresses (Ouellet et al., 1993; Hara et al., 2005; Kovacs et al., 2008; Brini et al., 2010; Cuevas-Velazquez et al., 2014). More recently, a reduction of stomatal density has been observed in transgenic *Arabidopsis* plants overexpressing *MtCAS31* through the interaction with the ICE1 transcription factor (Xie et al., 2012).

On the other hand, a positive relationship between the expression levels of DHNs transcripts or proteins and plant stress tolerance has been verified and usually more tolerant genotypes or cultivars have higher levels of DHNs than the less tolerant ones (Porat et al., 2004; Kosovà et al., 2010). Moreover, in different species heterologousexpression of *DHNs* genes, was found to confer tolerance to drought, cold and salinity (Hara et al., 2003; Rorat et al., 2006; Choudhury et al., 2007). For example, *Nicotiana tabacum* transgenic plants overexpressing *Citrus unshiu COR19* gene exhibited increased cold tolerance (Hara et al., 2003) as well as tolerance to water deficit and salinity was induced in transgenic plants of *Oryza sativa* overexpressing both *Hordeum vulgare HVA1* (Xu et al., 1996) and wheat *PMA80* and *PMA1959* genes (Cheng et al., 2002). Furthermore, an enhanced tolerance to osmotic stresses was also observed in *Arabidopsis* transgenic plants overexpressing the dehydrin DHN-5 of *Triticum durum* (Brini et al., 2010). Based on all these results, DHNs feature as useful tool to develop stress tolerance traits and this prompted us to enlarge information on this protein family.

In this context, we isolated and characterized a novel *DHN* gene from *oleaster* plants which is a prevalent component of evergreen vegetation that extends along the coastal and subcoastal areas in the Mediterranean, due to a high photosynthetic efficiency and drought tolerance (Bacchetta et al., 2003; Mulas et al., 2011). The gene was named *OesDHN* and includes an intron sequence within the S segment, a feature observed in other DHNs of both angiosperms and gymnosperms (Perdiguero et al., 2012, 2014; Jiménez-Bremont et al., 2013). The deduced amino acid sequence showed the typical lysine-rich K domain of angiosperm DHNs, and both homology and phylogenetic analyses categorized *Oes*DHN as an SK_2_-type DHN (Mundy and Chua, 1988; Close, 1996, 1997). Moreover, a nuclear target sequence, marked by the KKKK motif, was identified in the NLS region of *OesDHN*. Such predicted nuclear localization was then confirmed by laser confocal analysis, performed on 35S::*OesDHN*:GFP A 4-1 and 35S::GFP:*OesDHN* C11-5 *Arabidopsis* transgenic lines, showing the presence of *Oes*DHN protein inside the nucleus, This result is consistent with the nuclear localization detected for other DHNs, such as wheat DHN-5 and maize RAB17 proteins (Riera et al., 2004; Brini et al., 2007). Noteworthy, the nucleo-cytoplasmic distribution of DHNs is supposed to depend on protein phosphorylation status, which in turn can be controlled by stress conditions (Goday et al., 1994; Alsheikh et al., 2003; Brini et al., 2007; Mehta et al., 2009).

Additionally, *Oes*DHN protein was found also near the plasma membrane (Figure 6B). A similar localization has been observed for other SKn and KnS DHNs while it has been never reported for DHNs with the Y consensus (Hara et al., 2003; Xu et al., 2014). Thus, our results support the hypothesis that only the K segment is responsible for binding to membrane (Koag et al., 2009) and together with S segments exert a role in membrane protection (Graether and Boddington, 2014).

Concerning *OesDHN* expression pattern, it must be underlined that, although only at the transcript levels, for the first time the putative activity of a SK_2_-type DHN has been localized at the organ/tissue level (Graether and Boddington, 2014). In particular, we observed that in *oleaster* plants *OesDHN* is expressed in all organs, although at very different levels. Based on the suggested role of SK_2_-type DHNs (Kosova et al., 2010), such expression pattern could be related to tolerance traits of *oleaster* able to grow under multiple stress condition such as high salinity, high temperature and intense solar irradiance (Bacchetta et al., 2003; Mulas et al., 2011). Moreover, it must be mentioned that, although in some cases, expression is confined to specific organs or developmental stage, constitutive expression of *DHNs* has been commonly observed in both herbaceous and woody plants (Hara et al., 2001; Wang et al., 2007, 2011, 2014).

We also demonstrated that *OesDHN* expression is modulated during leaf development (i.e., it is higher in mature than in young leaves), and enhanced in the leaf and stem of *oleaster* plants exposed to mild water deficit. Under mild drought stress an increase of DHNs presence in leaf tissues was also observed through cyto-immunological analysis (Figure 2S) indicating that in *oleaster* plants defense mechanisms are enhanced at both transcriptional and post-transcriptional level. This result is in agreement with data in literature showing that DHNs highly accumulated in vegetative tissues of plants subjected to cellular dehydration, providing a protective mechanism (Brini et al., 2007, 2010). Notably, under mild drought stress the increased level of *OesDHN* transcripts and protein in the leaf was coupled to a well-preserved tissue organization, despite the water deficit. This result appears fully consistent with the role of space filler proposed for DHNs (Battaglia et al., 2008).

Finally, to confirm the involvement of *OesDH*N in plant stress response, its function was investigated by expressing it in *Arabidopsis* plants via the CaMV 35S promoter. We observed that these transgenic lines engineered to overexpress the *OesDH*N showed a better tolerance to osmotic stress than wild-type plants. This result fulfills other evidences showing that in transgenic plants *DHNs* expression is positively related to an increased tolerance to different stresses such as drought and salinity beside cold (Hara et al., 2003; Choudhury et al., 2007; Wang et al., 2011). In this context, it must be underlined that the involvement of DHNs in the protective system against osmotic stress is also supported by the up-regulation of *DHN* transcripts usually found upon salt or ABA treatment (Nylander et al., 2001; Sanchez-Ballesta et al., 2004). ABA is a phytohormone triggered by a multitude of abiotic stresses including drought, salinity, desiccation, and wounding and represents the major physiological signal that induces responses to drought and high salinity in plants (Gilmour et al., 1992; Lata and Prasad, 2011). Interestingly, our analysis of *OesDHN* promoter region highlighted the presence of different *cis-*elements involved in drought tolerance and the presence, among them, of an ABRE element suggests that *Oes*DHN could act as an ABA-responsive protein (Abe et al., 2003; Lata and Prasad, 2011).

In conclusion, we demonstrated that *Oes*DHN expression is induced by drought stress and is able to confer osmotic stress tolerance. Based on gene characterization and protein localization we also propose that, as reported for other dehydrins and LEA proteins (Dure, 1993; Riera et al., 2004; Brini et al., 2007) *Oes*DHN, interacting with distinct cellular components, could be involved in cell protection under water deficit and osmotic stress likely through an ABA-dependent pathway.

## Author contributions

AC, AM, LB, MV, and MB designed research; AC, AM, and LB performed research; AC, AM, LB, MV, and MB analyzed data and discussed results; AC and MB wrote the paper. All authors contributed to improving the paper and approved the final manuscript.

### Conflict of interest statement

The authors declare that the research was conducted in the absence of any commercial or financial relationships that could be construed as a potential conflict of interest.

## Supplementary material

Figure 1S **Schematic representation of the 35S::*****OesDHN*****, 35S::*****OesDHN*****:*****GFP*** **and 35S::*****GFP*****:*****OesDHN*** **binary expression vectors**. LB, left border; RB, right border; Pnos, nopaline synthase promoter; Kan, kanamycin gene; Tnos, termination signal of nopaline synthase gene; P35S, CaMV 35S promoter; *OesDHN* Olea, cDNA coding for putative dehydrin; Egfp, *Aequorea* victoria synthetic green fluorescent protein gene; T35S, termination signal of CaMV 35S gene; attB1, attB2, modified bacteriophage k attachment sites.

Figure 2S ***In situ*** **localization of dehydrin-like proteins on cross sections of mature leaves (length 2.5 ± 0.3 cm) grown in pots under control condition (A,B) and exposed to a SWC of 45% (C,D)**. n, nucleus; arrowhead indicate nuclear signal. Bars 25 μm **(C–E)**; 43 μm **(F)**; 28 μm **(A,C)**; 40 μm **(B,D)**; 90 μm **(E)**.
