# Supplementary figures and images for: A dehydrin gene isolated from feral olive enhances drought tolerance in Arabidopsis transgenic plants
Source: Front Plant Sci. 2015 Jun 30;6:392. doi: 10.3389/fpls.2015.00392 (PMC4485055; doi:10.3389/fpls.2015.00392)

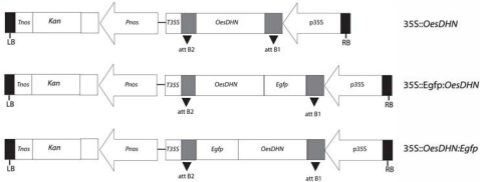

Figure 1S

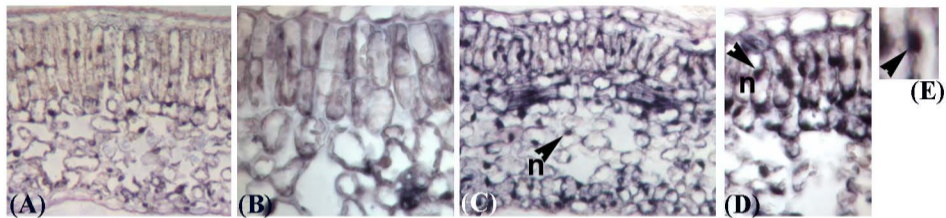

**Figure 2S**

Supplement: Figure 1S — Schematic representation of the 35S::OesDHN, 35S::OesDHN:GFP and 35S::GFP:OesDHN binary expression vectors. LB, left border; RB, right border; Pnos, nopaline synthase promoter; Kan, kanamycin gene; Tnos, termination signal of nopaline synthase gene; P35S, CaMV 35S promoter; OesDHN Olea, cDNA coding for putative dehydrin; Egfp, Aequorea victoria synthetic green fluorescent protein gene; T35S, termination signal of CaMV 35S gene; attB1, attB2, modified bacteriophage k attachment sites. [file Image1.PDF]

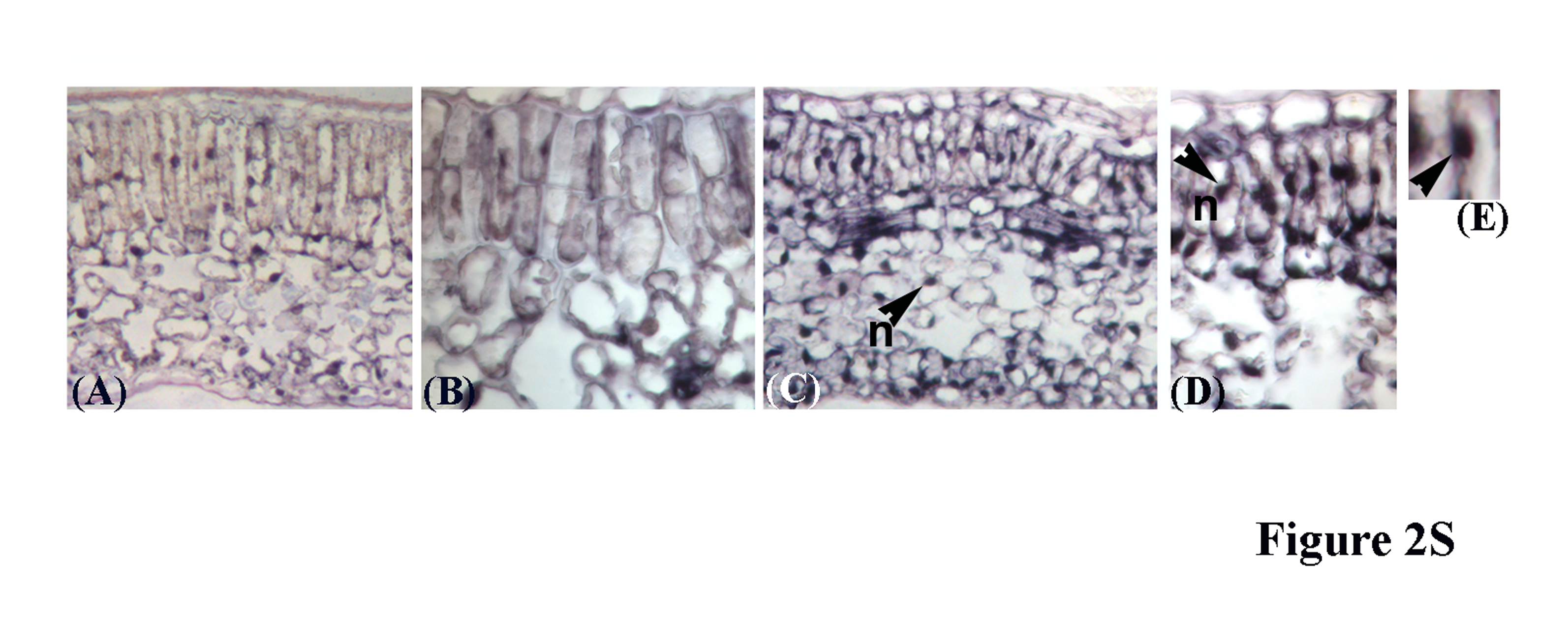

Supplement: Figure 2S — In situ localization of dehydrin-like proteins on cross sections of mature leaves (length 2.5 ± 0.3 cm) grown in pots under control condition (A,B) and exposed to a SWC of 45% (C,D). n, nucleus; arrowhead indicate nuclear signal. Bars 25 μm (C–E); 43 μm (F); 28 μm (A,C); 40 μm (B,D); 90 μm (E). [file Image2.TIF]
